# CircRNA_100269 is downregulated in gastric cancer and suppresses tumor cell growth by targeting miR-630

**DOI:** 10.18632/aging.101254

**Published:** 2017-06-27

**Authors:** Yan Zhang, Hao Liu, Wende Li, Jiang Yu, Jin Li, Zhiyong Shen, Gentai Ye, Xiaolong Qi, Guoxin Li

**Affiliations:** ^1^ Department of General Surgery, Nanfang Hospital, Southern Medical University, Guangdong Provincial Engineering Technology Research Center of Minimally Invasive Surgery, Guangzhou, China; ^2^ Guangdong Key Laboratory of Laboratory Animal, Guangdong Laboratory Animal Monitoring Institute, Guangzhou, China

**Keywords:** CircRNA_100269, MiR-630, gastric cancer, proliferation

## Abstract

Although CircRNA_100269 is a biomarker used to predict cancer recurrence, its expression and function in gastric cancer (GC) remain unknown. In this study, the expression of circRNA_100269 and its potential downstream miRNA targets were investigated. The molecular function and regulatory mechanism of circRNA_100269 in GC cell lines were also elucidated. The expression levels of circRNA_100269 and its linear isomer LPHN2 mRNA were found to be downregulated (p<0.01) in GC tissues. The target miRNA was predicted to be miR-630, whose expression was upregulated (p<0.01) and found to be negatively correlated with that of circRNA_100269 (r = −0.688) in GC tissues. Moreover, direct interaction of circRNA_100269 and miR-630 was confirmed through dual-luciferase assays. Overexpressing the circRNA_100269 plasmid inhibited cell proliferation (p<0.05). Furthermore, transfection of miR-630 mimics into cell lines overexpressing circRNA_100269 blocked the function of circRNA_100269 (p<0.05). Thus, circRNA_100269 level was downregulated in GC and correlated negatively with that of miR-630. Taken together, our results suggest that circRNA_100269 and miR-630 comprise a novel pathway that regulates proliferation of GC cells.

## INTRODUCTION

Gastric cancer (GC) is a common malignant tumor with the fourth highest occurrence among different cancers and is the third leading cause of death worldwide [1]. In 2013, 984,000 GC cases were diagnosed, of which, 841,000 resulted in death [2]. The incidence of GC is region specific—more than half of the patients diagnosed with GC live in east Asia countries, such as Japan and China [3, 4]. Although treatment options are available, mortality associated with GC remains high [5]. The mechanism underlying GC occurrence has not been fully understood. The incidence of GC is accompanied by a series of RNA and protein expression changes in cancer cells [6]. The molecular basis of GC progression must be elucidated to identify potential diagnosis markers and therapeutic targets.

Circular RNAs (circRNAs) are closed-loop RNAs produced through end-to-end joining of RNA transcription fragments during transcription [7]. Although circRNAs have been investigated for more than 40 years [8], they have not received significant attention until recent years. CircRNAs are abundant in many tissues [9, 10] and share the same sequence as the corresponding linear isomers but are formed through different splicing mechanisms [11]. Previous studies demonstrated that circRNAs are present in cancer cells. In certain cancers, circRNAs are aberrantly expressed in epithelial tumors, such as ovarian cancer [12], laryngeal cancer [13], and digestive system cancers [14–16], and in stromal tumors, such as gliomas [17]. In contrast to mRNAs, circRNAs are composed of a ring structure that is difficult to break down [18]. CircRNAs exhibit a strong regulatory function in carcinoma; in particular, circRNAs combine with microRNAs (miRNA) to regulate its function [19].

In our previous study, we found that circRNA_100269 is an independent predictor of early recurrence of stage III GC [20]. However, the mechanism of circRNA_100269 in cancer progression remains unknown. In the present study, we measured the expression levels of circRNA_100269 and its linear isomers in GC tissues to determine its expression pattern. The underlying mechanism of circRNAs was also explored by evaluating its downstream miRNA in GC cells. Moreover, we overexpressed circRNA_100269 in GC cell lines and performed cell proliferation experiments to elucidate its function.

## RESULTS

### CircRNA_100269 and LPHN2 were downregulated in GC tissues

**Figure 1:**
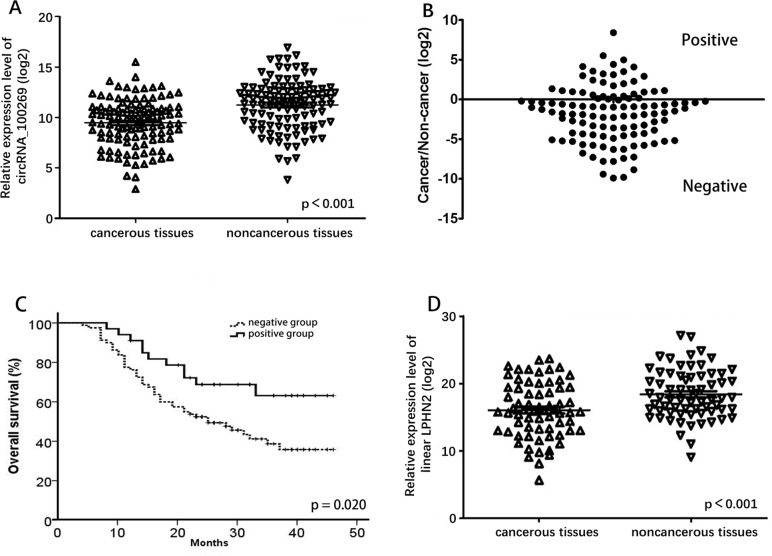
Relative expression level of circRNA_100269 and LPHN2 mRNA (**A**) Relative expression level of circRNA_100269 in 112 GC tissues and in paired adjacent normal tissues. (**B**) Cancer/non-cancer ratio of circRNA_100269 expression level in 112 GC tissues and in paired adjacent normal tissues. (**C**) The Kaplan-Meier survival analysis indicated that negative group has a worse overall survival compared to the positive group. (**D**) Relative expression level of LPHN2 mRNA in 67 GC tissues and in paired adjacent normal tissues. Error bars represent mean ± standard deviation (SD). P value was written in the figure corner.

qRT-PCR analysis was performed in 112 pairs of human GC specimens and their adjacent non-cancerous tissue samples to confirm circRNA_100269 expression in GC. CircRNA_100269 expression was reduced in 70.5% (79/112) of GC tissues (Fig. 1A) and was significantly lower in GC tissues than that in the corresponding adjacent non-cancerous tissues (P < 0.001). We divided all the cases into two groups according to the ratio of circRNA_100269 expression level in cancer tissues and adjacent tissues. In the negative group, the expression level of cancer tissues was lower than that of adjacent tissues, while the trend was opposite in the positive group (Fig. 1B). Results of Kaplan-Meier survival analysis indicated that the negative group has a worse overall survival compared to the positive group (Fig. 1C). Comparison of different clinical features indicated that the relative expression level of circRNA_100269 was correlated with histological subtypes and node invasion number assessed by pathological examination (Table 1).

**Table 1:** Association between circRNA_100269 expressions and clinicopathologic features

Clinicopathologic feature		Cases	CircRNA_100269 expression		P value
			Negative group	Positive group	
**Age**	Younger than 45 years	26	22	4	0.141
	Between 45 and 65 years	68	42	24	
	Older than 65 years	18	13	5	
**Gender**	Male	80	57	23	0.794
	Female	32	22	10	
**Histological subtypes**	Signet ring cell cancer	24	20	4	0.019
	Poorly differentiated adenocarcinoma	48	39	11	
	Other types	40	22	18	
**T stage**	T3 or lower	14	9	5	0.415
	T4a	76	52	24	
	T4b	22	18	4	
**Number of nodes invasion examined**	Less than 16	71	45	26	0.030
	16 or more	41	34	7	
**Lauren’s Classification**	Intestinal and mixed type	53	34	19	0.162
	Diffuse type	59	45	14	

We detected expression of linear LPHN2 mRNA, which is the linear isomer of circRNA_100269, in 67 randomly selected GC and adjacent tissues (Fig. 1D). Expression of LPHN2 in GC tissues was significantly lower than that in adjacent non-cancerous tissues (P < 0.001). Additionally, weak correlation was detected between circRNA_100269 and LPHN2 mRNA (Fig. S1A).

### CircRNA_100269 and miR-630 expressions were negatively correlated in GC tissues

**Figure 2:**
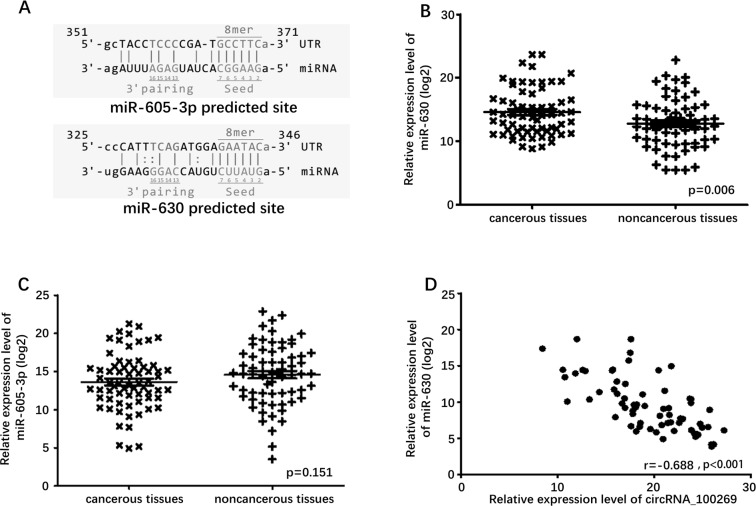
Predicted downstream miRNAs and their relative expression level (**A**) Predicted binding site of downstream miRNAs and circRNA_100269. (**B**) Relative expression level of miR-630 in 67 GC tissues and in paired adjacent normal tissues. (**C**) Relative expression level of miR-605-3p in 67 GC tissues and in paired adjacent normal tissues. (**D**) Correlation of expression between miR-630 and circRNA_100269. Error bars represent mean ± standard deviation (SD). P value was written in the figure corner.

The potential targets of circRNA_100269 were searched in bioinformatics databases via Target-Scan and miRanda to explore the underlying molecular mechanism. In addition, the potential binding sites of miR-605-3p and miR-630 in circRNA_100269 were predicted (Fig. 2A). To explore the relationship between circRNA_100269 and the predicted miRNAs in tissues, we examined the expression levels of miR-605-3p and miR-630 in GC and adjacent tissues. Expression of miR-630 was higher in GC tissues compared to adjacent tissues (P = 0.006), whereas expression of miR-605-3p was not significantly different (Fig. 2B and 2C). A significant negative correlation was found between the expression levels of miR-630 and circRNA_100269 (Fig. 2D), but miR-605-3p was not found to be correlated with circRNA_100269 (Fig. S1B).

### MiR-630 is a direct target of circRNA_100269

**Figure 3:**
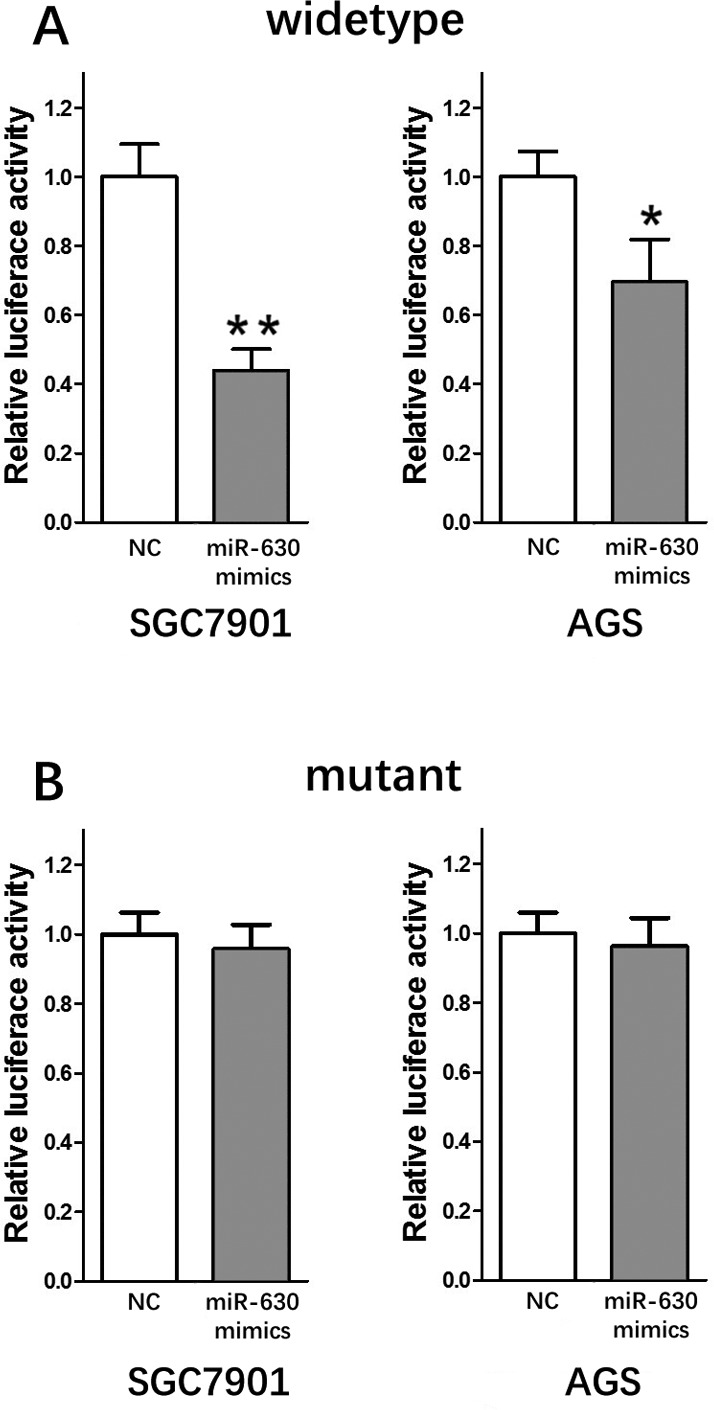
miR-630 is the direct target of circRNA_100269 (**A**) Fluorescence intensity was significantly reduced in the miR-630 mimics and wild-type dual-luciferase reporter plasmid-transfected group in the two GC cell lines. (**B**) Fluorescence showed no significant change in the miR-630 mimics and mutant dual-luciferase reporter plasmid-transfected group in the two GC cell lines. Error bars represent mean of three separate determinations ± standard deviation (SD). Asterisk indicates statistically significant changes: * (P < 0.05), ** (P < 0.01).

To determine whether miR-630 directly targets circRNA_100269, we constructed dual-luciferase reporter plasmids carrying a fragment of the mutant or wild-type circRNA_100269 sequence and the predicted miR-630 recognition site. A Dual-Luciferase Reporter Assay System was then adopted in random selected AGS and SGC7901 cells. Normalized fluorescence intensity of the reporter was significantly lower in cancer cells co-transfected with the circRNA_100269 segment and miR-630 mimics compared to controls (Fig. 3A). By contrast, no significant difference was detected between the control group and cells co-transfected with miR-630 mimics and mutant circRNA_100269 segment (Fig. 3B).

### CircRNA_100269 suppressed tumor growth in GC cell lines

**Figure 4:**
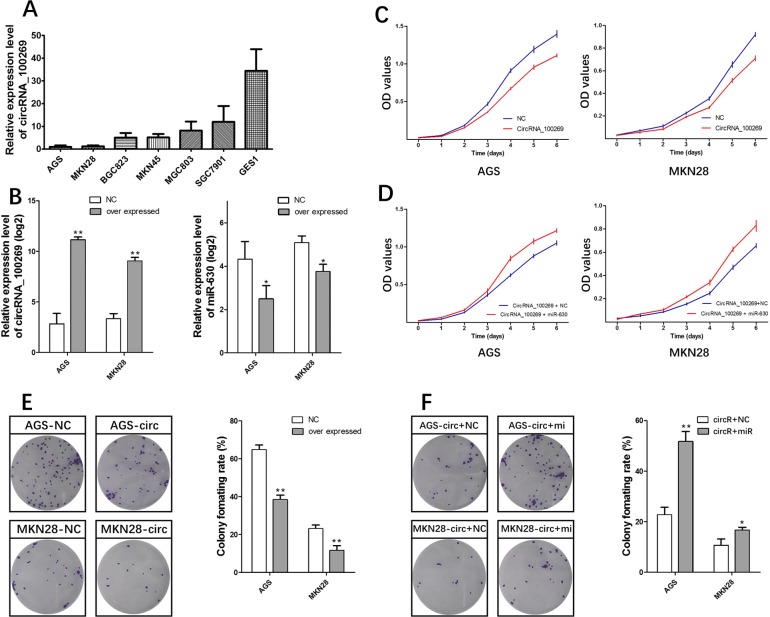
circRNA_100269-miR-630 axis mediated proliferation of GC cell lines (**A**) Endogenous circRNA_100269 expression in serial GC cell lines. (**B**) MiR-630 decreased by circRNA_100269 overexpression. (**C**) circRNA_100269 overexpression inhibits cell growth. (**D**) miR-630 can interrupt the cell growth inhibition function of circRNA_100269. (**E**) circRNA_100269 overexpression inhibits cell colony formation. (**F**) miR-630 can interrupt the inhibition function of cell colony formation of circRNA_100269. Error bars represent the mean of three separate determinations ± standard deviation (SD). Asterisk indicates statistically significant changes: * (P < 0.05), ** (P < 0.01).

We analyzed circRNA_100269 expression in human GC cell lines. Expression level of circRNA_100269 in AGS, MKN28, MKN45, BGC823, MGC803, and SGC7901 cells was significantly lower than that in the normal gastric mucosa cell line GES1 (Fig. 4A). We then performed gain of function assays by transfecting pcDNA3.1- circRNA_100269 or negative control into AGS and MKN28 cells, which showed the lowest expression among all the tested cell lines. Expression of circRNA_100269 significantly increased, which was concomitant with decrease in expression of miR-630 (Fig. 4B), in both cell lines transfected with pcDNA3.1- circRNA_100269, consistent with findings in tissue samples. We assessed the effect of circRNA_100269 on the proliferation of GC cells though CCK8 and cell formation assays. Overexpressing circRNA_100269 suppressed the proliferation of AGS and MKN28 cells (Fig. 4C and 4E). Our results indicated that circRNA_100269 was downregulated and inhibited the proliferation of GC cells.

### MiR-630 could suppress the function of circRNA_100269 in GC cells

If the effect of circRNA_100269 is specific, then the effect of overexpressing circRNA_100269 must be suppressed by co-expression of miR-630. Following this reasoning, miR-630 mimics, which can increase the levels of miR-630, were co-transfected with pcDNA3.1- circRNA_100269 into AGS and MKN28 cells. CCK-8 and cell formation assays were then performed. Growth rates significantly increased in groups with co-expressed circRNA_100269 and miR-630 mimics compared to that with co-expressed circRNA_100269 and NC mimics in both cell lines (Fig. 4D and 4F).

## DISCUSSION

CircRNAs exist widely in various organisms, including human cells [8]. CircRNAs and its linear RNA isomers are abundantly expressed in cells [21]. CircRNAs are organization- and disease-specific, and can thus be a potential biomarker for disease diagnosis [22]. CircRNAs exhibit many functions, e.g., as miRNA sponge, RBP sponge, or mRNA regulator. Some virus circRNAs can be translated into protein, but direct translation of eukaryotic circRNA has not been reported to date [23].

CircRNA_100269 is an exon circRNA transcript from the GRCh38.p7 fragment of chromosome 1, which is homologous to the protein coding gene LPHN2. LPHN2 encodes the latrophilin protein, a cell surface receptor, containing seven transmembrane segments [24].

LPHN2 is considered to be a downstream target of p53 and is aberrantly expressed in some tumors [25, 26]. In the present study, we confirmed that circRNA_100269 was downregulated in GC tissues. The linear isomer LPHN2 mRNA was also downregulated, indicating a possible link between circRNA_100269 and LPHN2 mRNA. However, the correlation between circRNA_100269 and LPHN2 mRNA was weak, indicating that their transcriptional regulation may occur differently. Exon circRNAs are formed upon cyclization of precursor RNA exons. Their expression levels are related not only to the quantity of precursor RNA, but also to the rate of the cyclization reaction.

A plethora of studies have been conducted on the roles of miRNAs in cancer. Various aberrantly expressed miRNAs have been found to be related to the progression and prognosis of GC [27, 28]. Most circRNA molecules contain the miRNA response element, which can bind to miRNAs. For example, circRNA_001569 has been found to promote the proliferation and invasion of colorectal cancer by targeting miR-145 [29]. circRNA ITCH could suppress esophageal squamous cell carcinoma via miR-7, miR-17, and miR-214 [30]. Based on this feature of circRNA, we predicted that miRNAs can interact functionally with circRNA_100269 and verified their expression relationship in GC tissues through dual-luciferase reporter assays. Result indicated that miR-630 and circRNA_100269 exhibit opposite expression trends in GC tissues and can interact directly in GC cell lines.

We sought to elucidate the direct biological function of the circRNA_100269-miR-630 axis in GC cell lines. First, two cell lines with the lowest expression of circRNA_100269 were chosen for overexpression experiments. We found that the level of miR-630 decreased significantly after overexpressing circRNA_100269. This suggested that circRNA_100269 may negatively regulate miR-630.

Next, the function of circRNA_100269 in proliferation of CRC cells was explored. Overexpressing circRNA_100269 in GC cells decreased the rate of cancer cell proliferation. Moreover, miR-630 decreased the function of circRNA_100269. These results strongly suggest that the circRNA_100269-miR-630 axis plays an important role in GC cell growth.

MiR-630 is one of the newly discovered miRNAs, and its role in cancer has gained increasing attention. MiR-630 is overexpressed in a variety of tumors [31, 32]. Further molecular studies have shown that miR-630 is related to cell invasion and metastasis [33]. Our previous studies also showed that miR-630 increased the radiosensitivity of colorectal cancer [34]. Thus, miR-630 is related to the biological behavior of cancer.

Our study has several limitations. Our studies were done primarily in cell-based assays. As such, the function of the circRNA_100269-miR-630 axis remains to be validated *in vivo*. Besides, although several miR-630 target genes were predicted by bioinformatics, it remains to be verified whether these represent bona fide targets in the context of GC. Further experimental analyses should be carried out to elucidate this.

In summary, we found that the expression levels of circRNA_100269 and its linear isomer were downregulated in GC tissues. Additionally, expression of the downstream target miR-630 was negatively correlated with circRNA_100269 expression. These results uncover a novel circRNA_100269-miR-630 signaling pathway involved in GC cell growth. Our findings highlight the diagnostic and therapeutic potential of these molecules in GC treatment.

## METHODS

### Population

A total of 112 patients diagnosed with GC were recruited in this study. All fresh tissues were collected between December 2012 and May 2015 during radical surgery at Nanfang Hospital of Southern Medical University. The samples were frozen in liquid nitrogen for 5 min and stored at −80°C. None of enrolled patients received chemotherapy, radiotherapy, or target therapy before radical surgery. The study protocol was approved by Institutional Review Board of Nanfang Hospital Southern Medical University. Informed consent was obtained from all patients involved in this study. All methods were performed in accordance with the relevant guidelines and regulations.

### Total RNA extraction

Frozen tissues were homogenized using Trizol reagent (Takara, Japan) to extract total RNA following the manufacturer’s instruction.

### qRT-PCR

cDNA was synthesized by reverse transcription using GoScript RT System (Promega, USA) and All-in-One miRNA Reverse Transcription Kit (GeneCopoeia, USA). qRT-PCR analysis was performed using GoTaq qPCR Master Mix (Promega, USA) and SYBR Green Human miRNA Assay Kit (GeneCopoeia, USA). The thermocycler programs were as follows: 95°C for 10 min and 40 cycles of 95°C for 30 s, 55°C annealing temperature for primer pairs for 30 s, and 72°C for 30 s. Each reaction was performed in triplicate. Reverse primers were designed to ensure the amplification of the head-to-tail splicing of circRNA.

### Target gene prediction

TargetScan (http://www.targetscan.org) and miRanda (http://www.microRNA.org) were used to predict potential circRNA_100269 targets. Although five miRNA targets were predicted, only two (namely, miR-630 and miR-605-3p) with expression in human tissues were followed up on. The binding site for circRNA_100269 was predicted to be at position 325–346 in miR-630 and at position 351–371 in miR-605-3p.

### Dual-Luciferase assay and vector construction

A circRNA_100269 segment (100bp) was synthesized with either mutant or wild-type seed region and cloned into the psiCHECK-2 vector (Applied Biosystems, USA). Five nucleotides in the seed region were mutated to obtain the mutant circRNA_100269 sequences. All cell lines were transfected using Lipofectamine 3000 (Invitrogen, USA). Cells (1×10^5^ cells/well) were transiently transfected with circRNA_100269 segment vector. Co-transfection with 20 nmol/L miR-630 mimics or control was then performed. Cells were harvested 48 h after transfection. The Dual Luciferase Reporter Assay System (Promega, Madison, USA) was used to detect luciferase activity.

Sequences of circRNA_100269 and 2,000 bp upstream and downstream segments of the circRNA_100269 gene location were synthesized and cloned into the pcDNA3.1 vector (Applied Biosystems, USA) to overexpress circRNA_100269.

### Cell culture

Human colorectal cancer cell lines, namely, AGS, MKN28, MKN45, BGC823, MGC803, SGC7901, and GES1, were purchased from the Cell Bank of Type Culture Collection (Shanghai City, China). All of these cell lines were maintained in RPMI 1640 medium containing 10% fetal bovine serum (HyClone, USA) in a humidified incubator at 37°C under 5% CO2.

### Cell proliferation assay

Cell proliferation was assayed using the Cell Counting Kit-8 (CCK8) assay and clone formation assay.

For CCK8 assay, the transfected cells were plated in 96-well plates (1000 cells/well). Cell proliferation was detected every 24 h according to the manufacturer’s protocol. Briefly, 10 μL of CCK 8 solution (KeyGene BioTECH, China) was added to each well and incubated at 37°C for 2 h. The solution was measured spectrophotometrically at 450 nm. Each group was analyzed three times.

For clone formation assay, the transfected cells were plated in six-well plates (200 cells/well). Cells were cultured for 2 weeks and stained with Giemsa after fixing with paraformaldehyde. The number of clones formed was counted, and the rate of colony formation ratio in each plate was calculated. Each group was analyzed three times.

### Statistical analysis

All statistical analyses were performed using SPSS 20.0 software (IBM, USA). Data were expressed as mean ± SD from at least three separate experiments. Differences between groups were analyzed using Student’s t test and Kruskal–Wallis test. The correlation between circRNA_100269 and miRNAs was analyzed using Pearson correlation test. P-value less than 0.05 was considered statistically significant.

## SUPPLEMENTARY MATERIAL



## Abbreviations

circRNA: circular RNA
miRNA: micro RNA
GC: gastric cancer
