# Supplementary material for: CircRNA_100269 is downregulated in gastric cancer and suppresses tumor cell growth by targeting miR-630
Source: Aging (Albany NY). 2017 Jun 27;9(6):1585–93. doi: 10.18632/aging.101254 (PMC5509457; doi:10.18632/aging.101254)
Supplement: Supplementary file 1 [file aging-09-1585-s001.pdf]

SUPPLEMENTARY MATERIAL

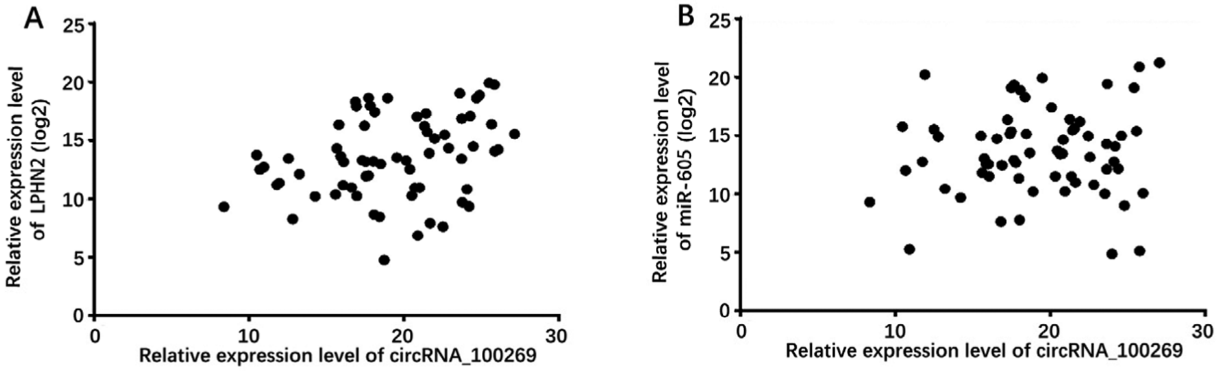

Figure S1. (**A**) Correlation of expression between linear LPHN2 and circRNA_100269. (**B**) Correlation of expression between miR-605 and circRNA_100269.
